# Correlation between Mechanical Properties and Processing Conditions in Rubber-Toughened Wood Polymer Composites

**DOI:** 10.3390/polym12051170

**Published:** 2020-05-20

**Authors:** Valentina Mazzanti, Lorenzo Malagutti, Andrea Santoni, Francesca Sbardella, Andrea Calzolari, Fabrizio Sarasini, Francesco Mollica

**Affiliations:** 1 Dipartimento di Ingegneria, Università degli Studi di Ferrara, via G. Saragat 1, 44122 Ferrara, Italy; lorenzo.malagutti@unife.it (L.M.); andrea.santoni@unife.it (A.S.); francesco.mollica@unife.it (F.M.); 2 Dipartimento di Ingegneria Chimica Materiali Ambiente, Sapienza-Università di Roma, Via Eudossiana 18, 00184 Rome, Italy; francesca.sbardella@uniroma1.it (F.S.); fabrizio.sarasini@uniroma1.it (F.S.); 3 ITW Test and Measurement Italy, Via Airauda 12, 10044 Pianezza (TO), Italy; andrea_calzolari@instron.com

**Keywords:** wood polymer composite, biocomposite, toughening agent, processing conditions, mechanical properties, starve feeding

## Abstract

The use of wood fibers is a deeply investigated topic in current scientific research and one of their most common applications is as filler for thermoplastic polymers. The resulting material is a biocomposite, known as a Wood Polymer Composite (WPC). For increasing the sustainability and reducing the cost, it is convenient to increase the wood fiber content as much as possible, so that the polymeric fraction within the composite is thereby reduced. On the other hand, this is often thwarted by a sharp decrease in toughness and processability—a disadvantage that could be overcome by compounding the material with a toughening agent. This work deals with the mechanical properties in tension and impact of polypropylene filled with 50 wt.% wood flour, toughened with different amounts (0%, 10%, and 20%) of a polypropylene-based thermoplastic vulcanizate (TPV). Such properties are also investigated as a function of extrusion processing variables, such as the feeding mode (i.e., starve vs. flood feeding) and screw speed. It is found that the mechanical properties do depend on the processing conditions: the best properties are obtained either in starve feeding conditions, or in flood feeding conditions, but at a low screw speed. The toughening effect of TPV is significant when its content reaches 20 wt.%. For this percentage, the processing conditions are less relevant in governing the final properties of the composites in terms of the stiffness and strength.

## 1. Introduction

An emerging class of materials that meets the urgent demand for plastics possessing a lower environmental impact are thermoplastics filled with fibers or particles of natural origin [1]. Among these bio-based materials, Wood Polymer Composites (WPCs) are the most widely used ones [2], with a global volume of 3.6 million tons in 2018 and an envisaged growth of up to 6.6 million tons by 2024, showing a compound annual growth rate of 10.6% during 2019–2024 [3]. The polymeric matrix is usually a commodity thermoplastic, such as polyethylene (PE) or polypropylene (PP), often coming from recycling [4,5,6], and the filler is comprised of wood particles or fibers, often originating from woodworking waste. In order to reduce the amount of plastics used, and hence the cost and environmental impact of the material, the greatest benefit is obtained with WPCs containing high quantities of natural filler. On the other hand, there are two main drawbacks that are linked to an increased wood content: brittleness [7] and a significant reduction in processability [8].

The main cause of embrittlement is poor interfacial adhesion between the hydrophilic wood and the hydrophobic polymer [9,10]. Suitable coupling agents improve the filler-matrix interface [11,12], but an additional possibility for reducing the brittleness is compounding WPC with toughening agents. Numerous studies have evaluated the addition of ethylene-propylene-diene rubber (EPDM) [7,9,13] and ethylene-propylene rubber (EPR) [12,14,15,16,17] in a wood-filled polypropylene system. In some papers, elastomers have also been functionalized with maleic anhydride, and this seems to be effective in increasing the impact strength due to a better compatibility with wood [7,9,12,14,16,17]. Furthermore, other impact modifiers have stimulated researchers’ interest, e.g., styrene-butadiene rubber (SBR) [18], ethylene-octene copolymer (EOC) [19,20], styrene-ethylene-butylene-styrene (SEBS) [9,21], and ethylene vinyl acetate (EVA) [22]. In general, in spite of a significant decrease in stiffness and strength, the presence of the elastomer improves the impact properties and the strain at break. For this reason, the main strategy is to optimize the relative amounts of wood filler and elastomer, in order to obtain the desired properties.

Although many positive results have been achieved, there is no general agreement on the effectiveness of toughening agents. The simultaneous presence of three phases, i.e., wood flour, polymeric matrix, and elastomer, does not always work properly because the elastomer particles may not provide a good interfacial adhesion with the thermoplastic matrix and/or the wood filler [18,21]. Indeed, the morphological structures within this multicomponent composite are quite complex [14,16]: elastomer and filler can be separately distributed within the polymeric matrix, but the elastomer may also encapsulate the filler [9,15,16]. As a result, the failure mechanisms are often difficult to understand. In fact, the topic is still debated and the complex interactions between the three phases and the resulting final mechanical properties mean that this issue is still not completely clear, yet stimulating to investigate. Moreover, these morphological differences may be influenced by several different factors [23], e.g., the size of the natural particles, the elastomer content, the polymer viscosity, the chemical interaction between the elastomer and the natural fibers, and the homogenization process. This, in turn, will heavily depend on the processing conditions.

Processing is indeed an important factor. A high content of natural filler reduces the processability: the viscosity increases at a higher wood content [24,25], so more energy is required for processing. Moreover, a high viscosity may induce localized heating by viscous dissipation, and this, in turn, may increase the risk of thermo-oxidative degradation [8]. Besides obvious deterioration of the natural filler, an additional consequence is the evolution of gaseous degradation products that will inevitably lead to voids within the material. On top of that, natural fibers tend to absorb moisture, as they are strongly hydrophilic, so without proper drying, water vapor may also contribute to the presence of point defects. The result is thus a higher porosity, which in turn reduces the mechanical properties of the finished product, irrespective of the use of coupling agents or other additives.

In the scientific literature, only a few studies have analyzed the effect of the extrusion conditions on the quality of WPC profiles [26,27]. Generally speaking, there is an improvement of the surface quality when the temperature and the length-to-diameter ratio of the die are increased and the extruder screw speed is reduced [28]. Moreover, a processing condition that may have a strong influence on the quality of these materials is the type of feeding. Single screw extruders are normally operated in a flood feeding condition: in this case, the screw channel is completely filled by the melting material. Therefore, the extrusion output is solely determined by the screw speed, and a relatively high pressure is developed along the screw channel. Less common is the use of single screw extruders in a starve fed condition: here, the flow rate is primarily determined by a volumetric or gravimetric feeder on the hopper, and it is thus independent of the screw speed [29]. This last type of feeding is often convenient when working with WPCs, because the pressure that is developed inside the barrel, and consequently the shear stresses to which the material is subjected, are lower. As a result, the danger of degradation is reduced. Moreover, by keeping the screw channel only partially filled, venting can also be performed through the inlet hopper, in addition to specific venting zones placed along the extruder. To the best of the authors’ knowledge, no study that combines the feeding type with the presence of a toughening agent has ever been conducted.

This work is concerned with a PP-based WPC filled with 50 wt.% of wood particles, which is further toughened with different percentages (0 wt.%, 10 wt.%, and 20 wt.%) of a commercial thermoplastic elastomer (Santoprene by EXXON Mobil) blended into it. The tensile and impact-tensile properties are studied as a function of the amount of toughening agent and the extrusion conditions in terms of the feeding type (i.e., starve vs. flood fed) and screw speed. Although the extrusion temperature is one of the main processing parameters, it will not be considered here: the thermal processing window of this material is quite small, being bound from below by the melting temperature of PP (i.e., 165 °C) and from above by the degradation temperature of the wood fibers (i.e., around 195 °C). For completeness, the morphology and fracture surface appearance will be analyzed using Scanning Electron Microscopy.

## 2. Materials and Methods

### 2.1. Materials

The materials that have been used in this study were obtained by blending a 70 wt.% PP-based WPC (PP CO 68/BZ) and a 30 wt.% PP-based WPC (PP 30 S) with a toughening agent. Details of the blending conditions are reported in the next subsection. Both WPC compounds were purchased from Plasticwood s.r.l. (Mazzantica di Oppeano, VR, Italy) and contained polypropylene and wood flour from white fir. The toughening agent was a commercial thermoplastic elastomer, i.e., Santoprene 201-55 (ExxonMobil, Irving, TX, USA), which is a PP-based thermoplastic vulcanizate (TPV). A complete characterization of both the 30 wt.% and 70 wt.% WPCs can be found in [24], while Leblanc [30] has analyzed the commercial TPV, performing xylene extraction at room temperature to evaluate the percentages in weight of the various components. In particular, Santoprene contains a significant amount of lubricant oil, i.e., 45.85 wt.%, seemingly working as a processing aid. Additional constituents of the TPV are EPDM (48.51 wt.%) and PP (5.64 wt.%). All blends studied in the present article had a fixed wood fiber content equal to 50 wt.%. All compositions and theoretical densities are listed in Table 1. The theoretical densities were obtained through mixture rule from the densities of the constituents, as declared by the producers. As the theoretical densities were obtained under idealized hypotheses, these were also intended to be the target densities for each material. It should be noted that the various materials display very little differences in terms of their theoretical densities.

### 2.2. Tensile Sample Preparation

For each material, the constituents (i.e., the 70 wt.% WPC compound, the 30 wt.% WPC compound, and the TPV at amounts appropriate for obtaining the compositions reported in Table 1) were fed into a single screw extruder (P.R.T. Service & Innovation, S. Agostino (FE), Italy), to obtain slabs with a 2 mm thickness and 50 mm width. The extruder had a screw diameter of 50 mm, a length over diameter ratio of 40, and was equipped with a breaker to improve compaction and a venting zone to help with degassing. Before extrusion, WPC pellets were dried at 80 °C overnight. A uniform temperature of 190 °C was set along the extruder barrel, while the die temperature was kept at 180 °C. For each material, two main extrusion conditions were studied, i.e., starve and flood fed. In the case of the flood fed condition, the screw speed was varied from 25 up to 100 RPM, while for the starve fed condition, only the 100 RPM screw speed was investigated. During all the extrusions, the die pressure was measured with a transducer (GEFRAN M22, ±0.25% full-scale accuracy).

### 2.3. Tensile Testing Procedure

Static mechanical characterization was performed in tension at room temperature using a universal testing machine (INSTRON 4467, Norwood, MA, USA). All tensile specimens (ISO 37-2011, type 1-A) were obtained by punch cutting from the extruded slabs and were loaded along the extrusion direction. All tensile tests were performed using a 500 N load cell and the crosshead speed was set at 2 mm/min. Digital imaging correlation (Dantec Dynamics, Skovlunde, Denmark) was used to evaluate the deformation until 0.2% for the determination of stiffness. Six dog bone specimens were tested for each material and processing condition.

From each tensile test sample, two 10 mm diameter disks were directly punch-cut to evaluate the apparent density and compare it with the theoretical value. The mass and dimensions of each sample were measured using a precision balance (Mettler AE240 with a resolution of 0.01 mg) and a digital micrometer (Mitutoyo 293 with a resolution of 2 μm), respectively.

### 2.4. Tensile-Impact Testing

Tensile-impact strength was investigated in accordance with ISO 8256 with a Type 1 specimen, i.e., 80 mm long, 10 mm wide, and 4 mm thick. Specimens were cut from 10 × 4 mm slabs that were obtained by extrusion through a dedicated die, using flood feeding at 25 RPM only, with the same extruder and in the same thermal conditions that were used for tensile testing specimens. The specimens were also double-notched with a dedicated knife with a 1 ± 0.05 mm radius and 45° ± 1° angle.

Tests were performed using an INSTRON 9450 Drop Tower system. The impact mass was set to 16 kg and the impact velocity to 2.9 m/s, with a related impact energy equal to about 69 J. The mass of the free grip (60 g) was considered in the calculations of the displacement and energy absorbed. The force-time plot for each specimen was acquired at a sampling frequency of 2 MHz by means of a piezoelectric cell (2.2 kN full scale). Three samples for each formulation were evaluated. The impact strength of notched specimens was calculated as the energy absorbed in breaking the specimen referred to the original cross-sectional area of the specimen at the notch, expressed in kJ/m^2^.

### 2.5. Scanning Electron Microscopy

The morphology of the fractured specimens after tensile-impact tests was evaluated by Scanning Electron Microscopy (SEM) by using a FEG-SEM Mira3 by Tescan. SEM analysis was performed on gold sputtered coated samples at an accelerating voltage of 10 kV. For each material, at least 30 images were acquired for ensuring the identification of a significant portion of material.

## 3. Results and Discussion

**Figure 1:**
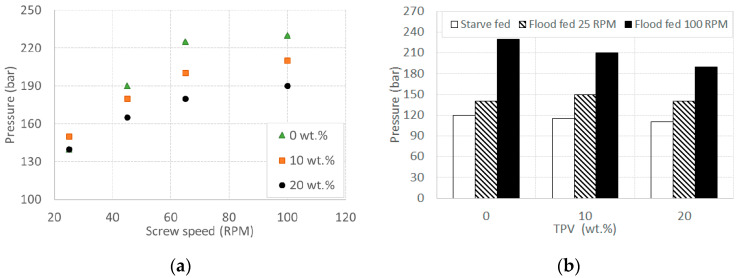
Die pressure (**a**) vs. screw speed for all materials in a flood fed condition at different screw speeds and (**b**) vs. thermoplastic vulcanizate (TPV) content in a starve fed condition at 100 RPM, flood fed condition at 25 RPM, and flood fed condition at 100 RPM.

Figure 1a displays the die pressure versus screw speed in a flood fed condition for all materials. As expected, the pressure increases with the screw speed, while it decreases significantly with an increasing TPV content. In Figure 1b, the die pressure vs. TPV content for the starve feeding condition at 100 RPM (white rectangles) is compared to the data relative to flood feeding at a 25 and 100 RPM screw speed. These two flood feeding conditions were chosen for comparison because the former has about the same flow rate, while the latter has the same screw speed. As it is clear from the figure, the die pressure in starve feeding mode is the lowest pressure because, in this case, only the final part of the extruder is completely filled with material, and the remaining part is only partially full. As a consequence, the pressure rise that is developed along the extruder barrel is much lower than that in flood feeding mode [29]. Interestingly, the trend in the pressure vs. TPV content that is observed in starve feeding is similar to the one for flood feeding at 100 RPM and also for all other flood feeding screw speeds that are shown in Figure 1a, with the exception of 25 RPM. The pressure decrease with an increasing TPV content can be easily justified on the basis of the large amount of lubricant that is present within the TPV: this promotes wall slip [30] and therefore reduces the pressure that is necessary for pumping the fluid through the die. In the case of flood feeding at 25 RPM, the die pressure seems to be independent of the TPV percentage. This may indicate that wall slip only becomes significant after a minimum value of the screw speed, i.e., around 25 RPM. Indeed, if the screw velocity decreases, the pressure decreases and so does the shear stress, and since the slip velocity primarily depends on the wall shear stress, wall slip can drop until being almost insignificant [31].

**Figure 2:**
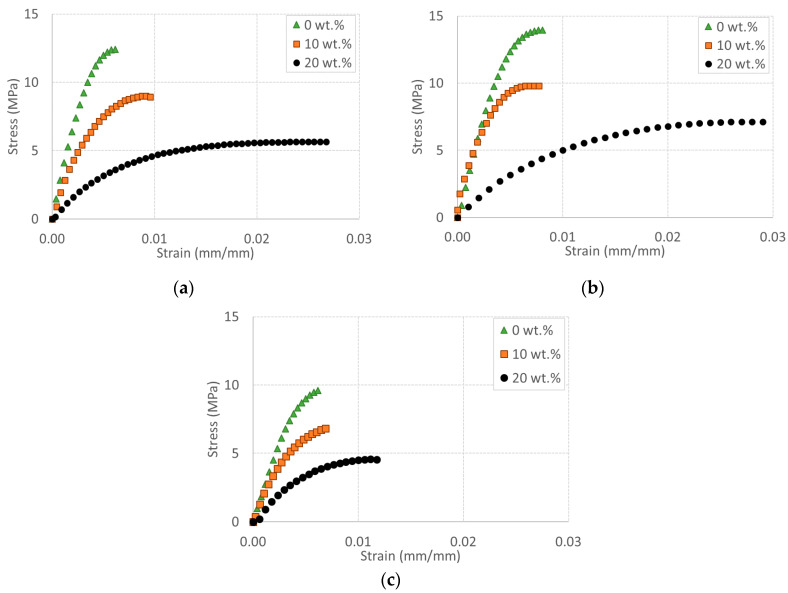
Stress vs. strain for all materials extruded in conditions of (**a**) starve feeding, (**b**) flood feeding at 25 RPM, and (**c**) flood feeding at 100 RPM.

Typical stress-strain curves for all materials are shown in Figure 2 as a function of processing conditions: (a) starve feeding at 100 RPM, (b) flood feeding at 25 RPM, and (c) flood feeding at 100 RPM. In general, a higher TPV content leads to a higher strain at break and a lower strength and stiffness. This is in agreement with the scientific literature [7,9]. Such effects are particularly significant at 20 wt.% TPV, where the strain at break reaches about 3%, except for the 100 RPM flood feeding condition. On the other hand, at a 10 wt.% TPV content, there is no increase in ductility, but both the strength and stiffness decrease.

**Figure 3:**
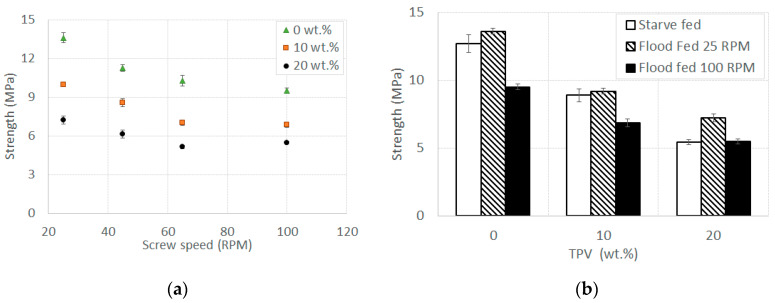
Tensile strength (**a**) vs. screw speed for all materials in a flood fed condition at different screw speeds and (**b**) vs. TPV content in starve fed conditions at 100 RPM, flood fed conditions at 25 RPM, and flood fed conditions at 100 RPM. Error bars indicate the standard deviation.

In Figure 3a, the tensile strength is plotted against the screw speed for all of the composites in flood feeding conditions. As the TPV content decreases, the strength increases. Interestingly, the strength decreases with an increasing screw speed. This behavior is quite surprising if compared to the die pressure readings displayed in Figure 1a: a higher die pressure should have a favorable effect on the final mechanical properties of the composite, because it increases the compaction of the material [32]. However, there is an important difference. Shakouri et al. [32] increased the die pressure, and thus the mechanical properties, by reducing the die thickness while maintaining the same screw speed; in our case, the situation is the opposite, i.e., the increase in the die pressure is strictly due to an increase of the screw speed without changing the geometry of the die. In Figure 3b, the tensile strength of all materials in starve feeding conditions at 100 RPM is compared to the strength of specimens produced in flood fed conditions at 25 and 100 RPM. Strength differences concerning the various processing conditions are statistically significant, as confirmed by ANOVA (*p* < 1%). Similar to Figure 3a, the strength also decreases with the TPV content in starve fed conditions. By analyzing Figure 3b in more detail, it can be observed that at a 0 wt.% and 10 wt.% TPV content, the materials obtained at 100 RPM in a flood fed condition display the highest strength drop, while the strengths of starve fed materials are similar to those of 25 RPM flood fed materials. Interestingly, at a 20 wt.% TPV content, the highest strength value is obtained for the 25 RPM flood fed material, while the starve fed material strength is more similar to that of the flood fed one at 100 RPM. However, such a difference, although statistically significant, is less than 2 MPa, so one can speculate that the processing conditions are less significant at a higher TPV content.

Strength reduction at a high screw speed can be explained by the occurrence of defects and porosity within the material originating from ineffective degassing when the screw speed increases. The formation of voids in the WPC structure during processing is a well-known phenomenon that cannot be completely avoided [33], and if it occurs too much, it can lead to a dramatic drop in strength. In fact, strength is particularly affected by localized defects because fracture occurs at the weakest point of a material, such as a discontinuity. Hence, degassing is one of the most important phases in WPC extrusion for eliminating porosity due to gaseous degradation products, humidity, or air entrapment. The venting zone that is present in the extruder used in this work may not work properly if the screw speed is too high: the material passes through this area too quickly and its effectiveness may be reduced.

Another possible reason for the drop of strength in conjunction with an increase of the screw speed can be related to the weak bond between natural fibers and the polymer-matrix interface. Areas of low adhesion, concurrently with a stress concentration zone, are often a valid justification for low mechanical properties and brittleness. The presence of a toughening agent in the composite changes the microstructure. During the compounding phase, all three components (i.e., polymer, wood, and TPV) arrange themselves in a structure that can be quite complicated [23]. Usually, two limit case structures can be formed: in the first one, the elastomer and filler are distributed separately within the polymer matrix, while in the second one, wood fibers can be encapsulated within the elastomer. It is important to note that during extrusion, both structures are formed, but one becomes prevalent over the other. If the elastomer surrounds the majority of wood fibers, the fiber-matrix interface is toughened and the possibility of severe crack development is reduced. For this reason, this last morphological structure should be preferable. As described by Pukánszky and Tudos [23], the final morphological structure depends on three factors: (1) the collision probability between the elastomer and fibers, which increases with the amount of elastomer; (2) the energy balance of the system, which, during mixing, leads to the creation of new surfaces and interfaces, thus favoring the embedded structure; and (3) the stability of the microstructure, influenced by the balance between the separating external forces (e.g., shear forces during the compounding) and the adhesion forces acting on the different components. This last factor, besides being the predominant one, can certainly vary according to the type of feeding. During extrusion, the shear forces act on both the reinforcement and the toughening agent. This is especially true when the extruder is in a flood fed condition and the flow rate is high. If these forces are greater than those of adhesion between the elastomer and the filler, the encapsulated wood can be easily separated and the elastomer dispersed in the polymer, thus leading to a less favorable microstructure.

**Figure 4:**
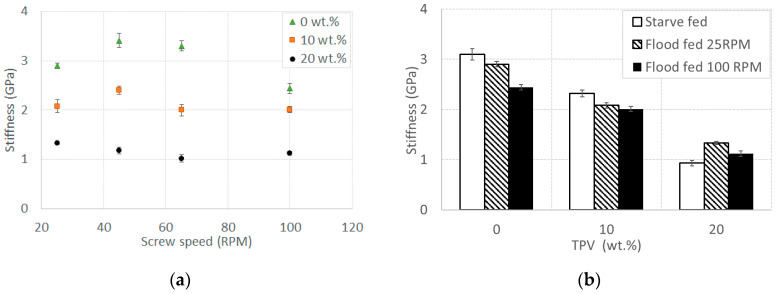
Tensile stiffness (**a**) vs. screw speed for all materials in a flood fed condition at different screw speeds and (**b**) vs. TPV content in a starve fed condition at 100 RPM, flood fed condition at 25 RPM, and flood fed condition at 100 RPM. Error bars indicate the standard deviation.

In Figure 4a, the stiffness as a function of the screw speed for all materials in a flood fed condition is shown. As noted before, the stiffness decreases as the content of TPV increases, while the screw speed seems to be almost unaffecting. This is consistent with the porosity being induced by the screw speed, as voids and defects usually have a smaller influence on the stiffness. In Figure 4b, the stiffness of all materials in a starve fed condition at 100 RPM is compared with those obtained in flood fed conditions at 25 and 100 RPM. Stiffness differences that are observed for each processing condition are statistically significant (ANOVA, *p* < 1%). As for the strength, when increasing the content of TPV up to 20 wt.%, the stiffness values at different processing conditions are closer to each other, as can be seen in both Figure 4a,b, even though such differences are statistically significant.

**Figure 5:**
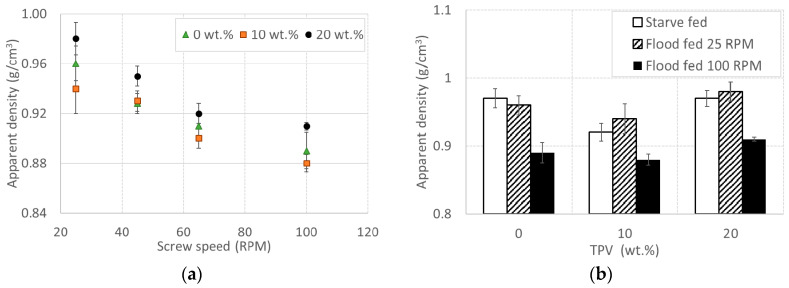
Apparent density (**a**) vs. screw speed for all materials in a flood fed condition and (**b**) vs. content of TPV in a starve fed condition at 100 RPM, flood fed condition at 25 RPM, and flood fed condition at 100 RPM. Error bars indicate the standard deviation.

To further verify the hypothesis that an increase in screw speed determines more voids and defects, the apparent density as a function of the TPV content at all screw speeds in a flood fed condition is shown in Figure 5a. It is quite clear that all of the apparent densities are lower than the target ones (Table 1), with the lowest values being those in the samples that were obtained at the highest screw speed and highest die pressure (Figure 1a). This last finding is particularly interesting, because the die pressure, although quite high, fails to achieve adequate compaction. Consistent results can be seen by comparing the apparent density at maximum and minimum screw speeds in flood fed conditions with the apparent density in a starve fed condition (Figure 5b). Despite the density values being quite close to each other, we found that the differences are statistically significant (ANOVA, *p* < 1%). The values of all the samples extruded in starve fed conditions are comparable to those for flood feeding at 25 RPM, but are much higher than those obtained in flood fed conditions at 100 RPM. These results are similar at any content of toughening agent and provide an indication about the correlation between the screw speed and presence of voids and defects.

In Table 2, the tensile-impact strengths, together with the maximum force and displacement at break, are listed. All materials were tested, but only in a flood feeding condition at a 25 RPM screw speed, since, in other conditions, excessive melt fracture of the extrudate was observed. The notched impact strength values indicate that the most significant toughening effect was obtained with 20 wt.% of TPV. This is in agreement with the results obtained with the tensile tests, where the materials with 10 wt.% TPV did not display a very different behavior from the material without toughening agent (Figure 2).

**Figure 6:**
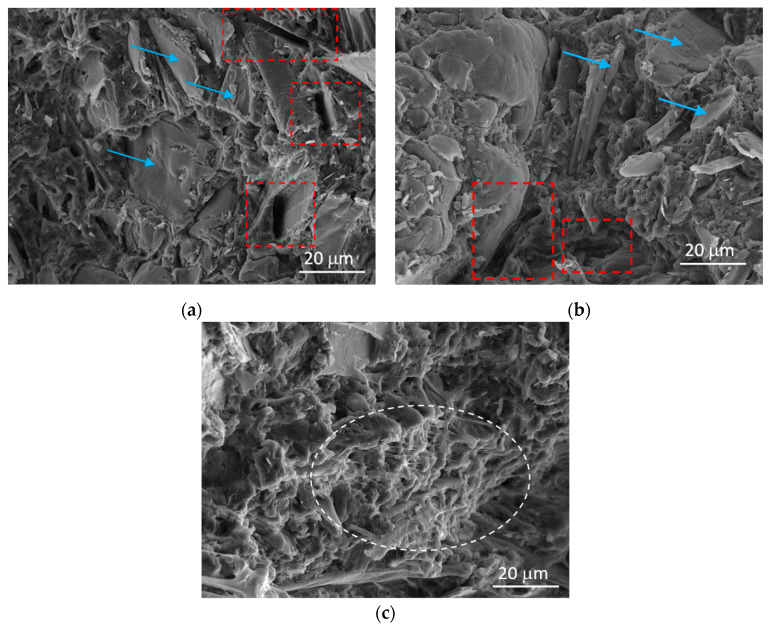
SEM micrographs of (**a**) 0 wt.%, (**b**) 10 wt.%, and (**c**) 20 wt.% in a flood fed condition at 25 RPM. The polymer ligaments can be identified by a white dotted ellipse, wood fibers by blue arrows, and sites of fiber pull-out by red dotted squares.

Figure 6 shows SEM scans of all the composites. Specimens were obtained from the fracture surfaces of high-speed tensile test samples. In all materials, wood fibers appear to be quite dispersed and embedded within the matrix, even though their interfacial adhesion seems quite poor, as confirmed by the presence of fiber pull-out and debonding phenomena. The sample with the highest amount of TPV has a morphology characterized by a much more ductile behavior, as evidenced by the presence of polymer ligaments (Figure 6c, dotted ellipse). This type of morphology can also be observed in other toughened WPCs [7,15].

As for static and high-speed tension tests, the SEM analysis also confirms that a 10 wt.% TPV percentage does not have a sufficiently appreciable toughening effect, even though it decreases the tensile strength and stiffness of the composites, due to the lower mechanical properties of TPV compared to PP. Another effect to consider is the potential reduction in crystallinity, as the impact modifiers tend to remain amorphous [34].

The toughness and ductility of short fiber reinforced composites are indeed affected by several diverse factors, such as the nature of the polymer matrix, the fiber volume fraction, the interfacial adhesion quality, and the fiber aspect ratio. The fiber amount has a significant influence on the reduction of elongation at break of the composites, because the regions close to the fiber ends are stress concentration points and constitute areas of poor adhesion where fibers are in contact with each other. In the present study, the wood fiber amount was quite high (50 wt.%) and the corresponding decrease in ductility and impact strength was only partially compensated for by the addition of the toughening agent, for which a higher fiber content (at least 20 wt.%) is needed to increase the dispersion of fibers, thus enhancing the energy that is needed to propagate the cracks [35]. Park and Balatinecz [34] reported a similar behavior for WPC based on PP reinforced with different amounts of wood fibers and EPDM. In particular, the effectiveness of EPDM in improving the elongation at break of EPDM-modified PP wood fiber composites was reduced with an increasing wood fiber amount, and at least 30 wt.% of EPDM was needed to counteract the negative effects imparted by 40 wt.% of wood fibers.

## 4. Conclusions

In this study a 50 wt.% PP-based WPC toughened with a TPV was investigated. Two different percentages of TPV were added to the compound in order to reduce the brittleness, and different processing conditions were evaluated.

The incorporation of TPV decreased the tensile stiffness and strength of PP-based composites for all TPV percentages, while the elongation at break only significantly increased with a 20 wt.% of TPV. These results were confirmed by dynamic tensile-impact tests, where composites modified with 20 wt.% of TPV displayed an almost doubled impact strength compared to unmodified samples. The strength of the composites was found to be deeply affected by the feeding conditions, being favored by a starve fed condition or flood fed condition, but with a reduced screw speed (i.e., not more than 25 RPM). The tensile stiffness was found to be relatively unaffected by the processing conditions. This behavior is ascribed to the enhanced formation of defects and porosity with an increasing screw speed, as assessed by a marked decrease in the apparent density of the samples. It can be concluded that a minimum amount of 20 wt.% of TPV is required to provide a significant enhancement of the composite toughness and ductility, but the material must be processed in a starve fed or flood fed condition with a low screw speed.

## Figures and Tables

**Table 1:** Description of the Wood Polymer Composite (WPC) compositions.

WPC Name	Wood (wt.%)	PP (wt.%)	TPV (wt.%)	Theoretical Density (g/cm^3^)
0 wt.%	50	50	0	1.07
10 wt.%	50	40	10	1.08
20 wt.%	50	30	20	1.09

**Table 2:** Tensile-impact strengths and their standard deviations in parentheses for all materials in a flood fed condition at 25 RPM.

WPC Name	Impact Strength (kJ/m^2^)	Force (N)	Displacement at Break (mm)
0 wt.%	9.56 (0.45)	751 (90)	0.57 (0.02)
10 wt.%	7.95 (1.25)	704 (55)	0.46 (0.08)
20 wt.%	18.70 (6.97)	524 (93)	1.52 (0.62)

## References

[1] Adekomaya O., Jamiru T., Sadiku R., Huan Z. (2016). A review on the sustainability of natural fiber in matrix reinforcement—A practical perspective. J. Reinf. Plast. Comp..

[2] Kumar V., Tyagi L., Sinha S. (2011). Wood flour-reinforced plastic composites: A review. Rev. Chem. Eng..

[3] Research and Markets. https://www.researchandmarkets.com/reports/4763051/wood-plastic-composites-market-global-industry?gclid=EAIaIQobChMIv_ae7o_C6AIVTeJ3Ch02QAlMEAAYAiAAEgL3e_D_BwE.

[4] Yeh S.K., Agarwal S., Gupta R.K. (2009). Wood-plastic composites formulated with virgin and recycled ABS. Comp. Sci. Tech..

[5] Sommerhuber P.F., Welling J., Krause A. (2015). Substitution potentials of recycled HDPE and wood particles from post-consumer packaging waste in Wood-Plastic Composites. J. Waste Manag..

[6] Ares A., Bouza R., Pardo S.G., Abad M.J., Barral L. (2010). Rheological, mechanical and thermal behaviour of wood polymer composites based on recycled polypropylene. J. Polym. Environ..

[7] Clemons C. (2010). Elastomer modified polypropylene-polyethylene blends as matrices for wood flour-plastic composites. Compos. Part A.

[8] Wang Q., Ou R., Shen X., Xie Y. (2011). Plasticizing cell walls as a strategy to produce wood-plastic composites with high wood content by extrusion processes. BioResources.

[9] Oksman K., Clemons C. (1998). Mechanical Properties and Morphology of Impact Modified Polypropylene—Wood Flour Composites. J. Appl. Polym. Sci..

[10] Mazzanti V., Pariante R., Bonanno A., Ruiz de Ballesteros O., Mollica F., Filippone G. (2019). Reinforcing mechanisms of natural fibers in green composites: Role of fibers morphology in a PLA/hemp model system. Comp. Sci. Tech..

[11] Bledzki A.K., Faruk O. (2003). Wood fibre reinforced polypropylene composites: Effect of fibre geometry and coupling agent on physico-mechanical properties. Appl. Comput. Math..

[12] Febrianto F., Hidayat W., Wistara N.J., Park S.H., Jang J.H., Lee S.H., Teramoto Y., Kondo T., Kim N.H. (2017). Influence of impact modifier-coupling agent combination on mechanical properties of wood flour-reinforced polypropylene composite. J. Fac. Agric..

[13] Meekum U., Khongrit A. (2018). Toughening of wood-plastic composites based on silane/peroxide macro crosslink poly(propylene) systems. BioResources.

[14] Várdai R., Lummerstorfer T., Pretschuh C., Jerabek M., Gahleitner M., Pukánszky B., Renner K. (2019). Impact modification of PP/wood composites: A new approach using hybrid fibers. eXPRESS Polym. Lett..

[15] Keledi G., Sudár A., Burgstaller C., Renner K., Móczó J., Pukánszky B. (2012). Tensile and impact properties of three-component PP/wood/elastomer composites. eXPRESS Polym. Lett..

[16] Sudár A., Rennera K., Móczóa J., Lummerstorferc T., Burgstaller C., Jerabekc M., Gahleitnerc M., Doshevc P., Pukánszky B. (2016). Fracture resistence of hybrid PP/elastomer/wood composites. Compos. Struct..

[17] Sudár A., Burgstaller C., Renner K., Móczó J., Pukánszky B. (2014). Wood fiber reinforced multicomponent, multiphase PP composites: Structure, properties, failure mechanism. Comp. Sci. Tech..

[18] Kakroodi A.R., Leduc S., González-Núñez R., Rodrigue D. (2011). Mechanical Properties of Recycled Polypropylene/SBR Rubber Crumbs Blends Reinforced by Birch Wood Flour. Polym. Polym. Comp..

[19] Kazemi Y., Cloutier A., Rodrigue D. (2013). Mechanical and morphological properties of wood plastic composites based on municipal plastic waste. Polym. Comp..

[20] Sombatsompop N., Yotinwattanakumtorn C., Thongpin C. (2004). Influence of Type and Concentration of Maleic Anhydride Grafted Polypropylene and Impact Modifiers on Mechanical Properties of PP/Wood Sawdust Composites. J. App. Polym. Sci..

[21] Sharma R., Maiti S.N. (2015). Effects of crystallinity of polypropylene (PP) on the mechanical properties of PP/styrene-ethylenebutylene-styrene-g-maleic anhydride (SEBS-g-MA)/teak wood flour (TWF) composites. Polym. Bull..

[22] Ghahri S., Najafi K., Mohebby B., Tajvidi M. (2010). Impact Strength Improvement of Wood Flour–Recycled Polypropylene Composites. J. Appl. Polym. Sci..

[23] Pukánszky B., Tudos F. (1990). Ternary Composites of Polypropylene, Elastomer and Filler: Analysis of Phase Structure Formation. Polym. Comp..

[24] Mazzanti V., Mollica F., El Kissi N. (2016). Rheological and mechanical characterization of polypropylene-based wood plastic composites. Polym. Comp..

[25] Mazzanti V., Mollica F. (2019). Rheological behavior of wood flour filled poly (lactic acid): Temperature and concentration dependence. Polym. Comp..

[26] Soury E., Behravesh A.H., Jam N.J., Haghtalab A. (2012). An experimental investigation on surface quality extruded wood-polypropylene composite. J. Adv. Mater. Res..

[27] Cai J., Jia M., Xue P., Ding Y., Zhou X. (2013). The effect of processing conditions on the mechanical properties and morphology of self-reinforced wood-polymer composite. Polym. Comp..

[28] Ritter S., Poszvek M., Riegler M., Sykacek E. (2016). Process analysis using multivariate regression models exemplified by WPC processing with a single-screw extruder. Int. Wood Prod. J..

[29] Wilczyński K.J. (2018). Experimental and theoretical study on screw filling in starve fed single screw extruders. Int. Polym. Process..

[30] Leblanc J.L. (2007). Nonlinear viscoelastic characterization of molten thermoplastic vulcanizates (TPV) through large amplitude harmonic experiments. Rheol. Acta.

[31] Mazzanti V., Mollica F. (2017). Pressure dependent wall slip of wood flour filled polymer melts. J. Non Newton. Fluid..

[32] Shakouri E., Behravesh A.H., Zolfaghari A., Golzar M. (2009). Effect of die pressure on mechanical properties of wood-plastic composite in extrusion process. J. Thermoplast. Compos. Mater..

[33] Klyosov A. (2007). Wood-Plastic Composites.

[34] Park B.D., Balatinecz J.J. (1997). Mechanical Properties of Wood-Fiber Toughened lsotactic Polypropylene Composites. Polym. Comp..

[35] Boldizar A., Klason C., Kubát J., Näslund P., Sáha P. (1987). Prehydrolyzed Cellulose as Reinforcing Filler for Thermoplastics. Int. J. Polym. Mater..

